# Run-Reversal Equilibrium for Clinical Trial Randomization

**DOI:** 10.1371/journal.pone.0128812

**Published:** 2015-06-16

**Authors:** William C. Grant

**Affiliations:** 1 Department of Economics, James Madison University, Harrisonburg VA, United States of America; 2 Visiting Faculty, Duke Clinical Research Institute, Durham NC, United States of America; Cardiff University, UNITED KINGDOM

## Abstract

In this paper, we describe a new restricted randomization method called run-reversal equilibrium (RRE), which is a Nash equilibrium of a game where (1) the clinical trial statistician chooses a sequence of medical treatments, and (2) clinical investigators make treatment predictions. RRE randomization counteracts how each investigator could observe treatment histories in order to forecast upcoming treatments. Computation of a run-reversal equilibrium reflects how the treatment history at a particular site is imperfectly correlated with the treatment imbalance for the overall trial. An attractive feature of RRE randomization is that treatment imbalance follows a random walk at each site, while treatment balance is tightly constrained and regularly restored for the overall trial. Less predictable and therefore more scientifically valid experiments can be facilitated by run-reversal equilibrium for multi-site clinical trials.

## Introduction

In many areas of scientific inquiry, experts believe that randomized experiments are the "gold standard" for hypothesis testing [1] However, faith in the scientific validity of randomized experiments is misplaced whenever investigators are able to predict treatments. In the field of medicine, in particular, investigators are able to predict treatments in many clinical trials, and potential bias threatens an alarming proportion of all clinical experiments [2]. The game-theoretic concept of Nash equilibrium offers new ways to design randomized clinical trials in order to minimize treatment predictability.

In this paper, we introduce a method called run-reversal equilibrium (RRE) for randomizing patients in multi-site clinical trials. The procedure is appropriate as long as treatment balance is needed for the overall trial but site-specific treatment balance is needed less or not at all. Imagine a game where the clinical trial statistician, who seeks scientific validity, chooses a sequence of medical treatments, while simultaneously each investigator chooses a treatment forecasting strategy. We call a Nash equilibrium of such a treatment prediction game a run-reversal equilibrium. RRE reflects how the treatment history at a particular site is correlated with the treatment imbalance for the overall trial, and the statistician’s RRE strategy (i.e. the randomization procedure) makes treatments unpredictable from the perspective of each clinic’s investigator, while simultaneously achieving some given level of treatment balance for the overall trial.

This particular type of Nash equilibrium has not previously been applied to the context of clinical experiments, but other game theory concepts have illuminated this subject for quite some time. Blackwell and Hodges’ seminal paper [3] from the statistics literature dates to 1957. Coincidentally, separate work by the same David Blackwell has proved to be extremely influential in the field of game theory concerned with correlated equilibrium [4]. More recent game-theoretic work has proved theoretically insightful for understanding the nature of clinical trials [5]. Run-reversal equilibrium, however, is more realistic to implement because it requires no prior information regarding the distribution of patient prognosis.

The paper is organized as follows. The Methods section presents the general setup of treatment prediction games: strategies, information partitions, and payoffs. Clinical investigators are assumed to forecast treatments as accurately as possible, meaning in game theory terminology that their payoffs are increasing in treatment predictability. In contrast, the statistician seeks to minimize potential bias by choosing an unpredictable sequence of treatments. A specific example of a 3-site game and its run-reversal equilibrium are described in the Results and Discussion section. The next results describe run-reversal equilibria for clinical trials with four sites, five sites, and six sites. Finally, the Conclusions section identifies the conditions under which our RRE randomization procedures are appropriate for use in real-world clinical trials.

## Methods

### General Framework for Treatment Prediction Games

#### Players and strategies

The set of players N for a multi-site clinical trial consists of one clinical trial statistician, denoted by stat, and some number of investigators, one investigator for each site participating in the experiment. The statistician’s strategy set reflects typical clinical trial protocols described by Berger et al. [6]: first, the set of acceptable treatment sequences are defined; and second, a probability distribution is chosen and used to select one sequence. Most clinical trials constrain the overall treatment imbalance, which means that the set of acceptable treatment sequences includes only sequences where the patient counts of alternative treatments are not too different [7], [8]. That is, a ceiling is placed on the difference between the number of times that one treatment is assigned and the number of times that an alternative treatment is assigned. Sometimes, clinical trial practitioners also seek balance with respect to prespecified subject characteristics such as gender [9]. In that case, the treatment prediction game would involve each investigator observing a history of previous treatments and previous genders. The use of a treatment imbalance constraint reflects the statistical need for hypothesis-testing power, the clinics’ operational capacity for administering treatments, and/or the ethical need to limit the fraction of placebo treatments. In our view, each acceptable treatment sequence is equivalent to a pure strategy for the statistician, and the probability distribution over the set of acceptable treatment sequences is a mixed strategy for the statistician. We will denote the set of acceptable treatment sequences by C_stat_, and a particular treatment sequence from this set as c_stat_. The probability distribution which the statistician uses to select a particular sequence is denoted by μ_stat_.

Suppose that there are just two treatments, one active treatment T, and one glycerin control treatment G. Each strategy for an investigator is a forecast plan, consisting of a set of probabilistic forecasts, one active treatment forecast for each possible investigator information set. Each investigator’s information partition is defined on the set of treatment histories which could be unmasked at that investigator’s site. Each site investigator observes the treatment at his particular site after it has been administered because of intentional and/or unintentional unmasking. Unintentional unmasking occurs when the act of administering the treatment gives away its identity, whereas intentional unmasking occurs for open-label trials where, for example, safe administration of the treatment requires that the investigator learns the treatment identity after it has been assigned. Whether intentional and/or unintentional, unmasking allows each investigator to condition each forecast for the next patient’s treatment on his observation of the previous patients’ treatments. Let h^t^_i_(c_stat_) be the particular history of treatments preceding t and unmasked to the investigator at site i when the statistician has selected treatment sequence c_stat_; we denote each history h^t^_i_(c_stat_) as h^t^_i_ for short. Let p_i_|h^t^_i_ be investigator i's probabilistic forecast that the t^th^ patient will be assigned to active treatment, conditional on history h^t^_i_. A collection of probabilistic forecasts, one for each treatment history i could observe (i.e. a forecast plan) is denoted as (p_i_|h)_hϵHi_, where H_i_ is the set of all possible treatment histories that i could encounter. A particular forecast plan (p_i_|h)_hϵHi_ constitutes a particular strategy for investigator i and is denoted (p_i_|h) for short.

#### Payoffs

We assume that the clinical trial statistician seeks to minimize excess predictability, while each investigator makes forecasts which are as accurate as possible in terms of Bayesian rationality. If investigators can do better than choosing forecasts equal to the unconditional prior treatment probability (i.e. the allocation ratio), then there is excess predictability. In the clinical trials literature, excess predictability is typically expressed in terms of the expected rate of correct predictions minus the allocation ratio [2], [3], [6], [10], [11], [12], [13], [14], [15]. We choose to express predictability in a very similar way, but we diverge from the standard treatment of each prediction as 100% correct or 100% incorrect. Instead, because investigators can make probabilistic forecasts, we express excess predictability in terms of the probability forecasted to the correct prediction minus the allocation ratio. Suppose that the two treatments, active treatment T and glycerin control treatment G, are to be assigned equally, so that the allocation ratio equals 1/2. Let T^t^_i_ be an indicator variable for active treatment where T^t^_i_ = 1 if the treatment sequence selected by the statistician assigns active treatment for the t^th^ patient at site i, and T^t^_i_ = 0 if control is assigned. The excess predictability at site i resulting from a mixed strategy μ_stat_ is equal to:

$$EP_{i}=\;E\left\{\sum_{t}\left[p_{i}^{t}\cdot T_{i}^{t}+\left(1-p_{i}^{t}\right)\cdot \left(1-T_{i}^{t}\right)-\frac{1}{2}\right]\right\}$$ (1)

where the expectation is taken with respect to μ_stat_. If investigators predict the correct treatment with probability greater than the allocation ratio (in this case, 50%), then there is positive excess predictability. If all forecasts are equal to the allocation ratio, or if greater-than-50% probability is placed on the incorrect treatment just as much as it is placed on the correct treatment, then excess predictability equals zero. Eq (1) is consistent with the preponderance of literature (cited above) treating treatment predictability as the expected rate of correct predictions minus the allocation ratio, but (1) is a more flexible way to represent predictability because it allows for forecasts to be probabilistic. For the clinical trial across all sites, the excess predictability from μ_stat_ equals the summation of (1) across all sites:

$$EP=\;\sum_{t}EP_{i}$$ (2)

The statistician’s payoff U_stat_ is decreasing in excess predictability and is written as:

$$U_{stat}=statistician\;payoff=U_{stat}\left(EP\right)$$ (3)

We choose to leave Eq (3) in a general form because the specific functional form of U_stat_(EP) is unclear and, more importantly, is not necessary for our purposes. One possibility is an absolute value function such as that found in [3], while another alternative is a quadratic form similar to a standard a quadratic loss or a calibration score such as that found in [16]. The only quality of (3) relevant for the treatment prediction games described here is that U_stat_(EP) is maximized if EP equals zero. When this is the case, there is zero potential for treatment predictability to create selection bias. That is, EP equaling zero implies that the treatment variable is statistically independent from other factors selected by the investigator to affect the dependent variable.

Each investigator i’s payoff is increasing in EP_i_, and we again will use a general form for each investigator’s payoff:

$$U_{i}=investigator\;i^{\prime }s\;payoff=U_{i}\left(EP_{i}\right)$$ (4)

Each investigator prefers more predictability in order to make treatments dependent on other factors selected by the investigator which affect the dependent variable. Any of the motivations and behaviors described in Section 2 help justify this assumption.

#### Run-reversal equilibrium

Let M(C_stat_) be the set of all probability distributions over the treatment sequences C_stat_. Also, for any forecast profile ((p_i_|h))_i_, we let ((p_-i_|h))_-i_ be the list of forecast plans for all investigators except i. A run-reversal equilibrium is a Nash equilibrium of a treatment prediction game, consisting of the statistician’s choice of randomization procedure μ_stat_* and the profile of investigator forecast plans ((p_i_|h)*)_i_ such that

$$\begin{array}{l} U_{stat}\left(\mu_{stat}*,\;{\left(\left(p_{i}\;|\;h\right)*\right)}_{i}\right)\geq U_{stat}\left(\mu_{stat},\;{\left(\left(p_{i}\;|\;h\right)*\right)}_{i}\right)\forall \;\mu_{stat}\in \text{M}\left(C_{stat}\right)\\ \text{and}\\ U_{i}\left(\mu_{stat}*,\left(p_{i}\;|\;h\right)*,\;{\left(\left(p_{-i}|\;h\right)*\right)}_{-i}\right)\geq U_{i}\left(\mu_{stat}*,\left(p_{i}\;|\;h\right),\;{\left(\left(p_{-i}|\;h\right)*\right)}_{-i}\right)\;\forall \;\left(p_{i}\;|\;h\right)\; \end{array}$$ (5)

Treatment prediction games and the corresponding run-reversal equilibria satisfying (5) can be interpreted along lines explained by [17]. The statistician deliberately introduces randomness into his behavior, so that μ_stat_ is a literal device (a deck of sequence-labeled cards, or a computer program are examples of such actual devices). Simultaneously, μ_stat_ determines investigator beliefs which investigators use to determine what forecast profile is optimal in terms of (5). Given μ*, each investigator’s RRE forecasting strategy comes from setting forecasts equal to the Bayesian posterior probabilities derived from μ*.

## Results and Discussion

### A Clinical Trial Game with Three Clinical Sites

#### The statistician’s strategies with three clinical sites

The first example in this paper involves three clinical sites, where each site enrolls patients sequentially one at a time. We begin with a three-site trial because this is the smallest number of sites where the players’ information will be imperfectly correlated. Section 5 describes 4-, 5-, and 6-site trials. The investigators at site x, site y, and site z are denoted by their site names, so that N equals {stat, x, y, z}. At the beginning of the game, the statistician chooses a treatment assignment sequence which dictates the sequence of trios of treatments, from the trio of first patients arriving at the three sites, through the trio of last patients arriving at the three sites. The statistician’s pure strategy set contains all treatment sequences satisfying some exogenous balance constraint, and the statistician chooses a randomization from the set of all probability distributions over these sequences.

The exogenous treatment balance constraint for this example is very strict: the overall trial treatment imbalance must be less than or equal to one, upon completion of each treatment trio, where a treatment trio consists of the t^th^ patient at site X, the t^th^ patient at site Y, and the t^th^ patient at site Z. Consider the game with just two treatment trios (i.e. with two patients enrolled at each of the three sites). Obviously, nearly every real-world trial involves more than six total patients. Our two-trio game solution can readily be extended to longer trials. A 100-trio trial (i.e. 300 patient trial with 100 patients enrolled at each of the three sites), for instance, can be viewed as 50 separate repetitions of our two-trio game. With two treatments T and G, the statistician’s choice set is

$$\begin{array}{l} {\text{C}}_{\text{stat}}=\{{\text{G}}^{\text{1}}{}_{\text{x}}{\text{G}}^{\text{1}}{}_{\text{y}}{\text{T}}^{\text{1}}{}_{\text{z}}{\text{T}}^{\text{2}}{}_{\text{x}}{\text{T}}^{\text{2}}{}_{\text{y}}{\text{G}}^{\text{2}}{}_{\text{z}}{\text{,G}}^{\text{1}}{}_{\text{x}}{\text{G}}^{\text{1}}{}_{\text{y}}{\text{T}}^{\text{1}}{}_{\text{z}}{\text{G}}^{\text{2}}{}_{\text{x}}{\text{T}}^{\text{2}}{}_{\text{y}}{\text{T}}^{\text{2}}{}_{\text{z}}{\text{,G}}^{\text{1}}{}_{\text{x}}{\text{G}}^{\text{1}}{}_{\text{y}}{\text{T}}^{\text{1}}{}_{\text{z}}{\text{T}}^{\text{2}}{}_{\text{x}}{\text{G}}^{\text{2}}{}_{\text{y}}{\text{T}}^{\text{2}}{}_{\text{z}},\;\\ {\text{T}}^{\text{1}}{}_{\text{x}}{\text{T}}^{\text{1}}{}_{\text{y}}{\text{G}}^{\text{1}}{}_{\text{z}}{\text{G}}^{\text{2}}{}_{\text{x}}{\text{,G}}^{\text{2}}{}_{\text{y}}{\text{T}}^{\text{2}}{}_{\text{z}}{\text{,T}}^{\text{1}}{}_{\text{x}}{\text{T}}^{\text{1}}{}_{\text{y}}{\text{G}}^{\text{1}}{}_{\text{z}}{\text{T}}^{\text{2}}{}_{\text{x}}{\text{G}}^{\text{2}}{}_{\text{y}}{\text{G}}^{\text{2}}{}_{\text{z}}{\text{,T}}^{\text{1}}{}_{\text{x}}{\text{T}}^{\text{1}}{}_{\text{y}}{\text{G}}^{\text{1}}{}_{\text{z}}{\text{G}}^{\text{2}}{}_{\text{x}}{\text{T}}^{\text{2}}{}_{\text{y}}{\text{G}}^{\text{2}}{}_{\text{z}},\;\\ {\text{G}}^{\text{1}}{}_{\text{x}}{\text{T}}^{\text{1}}{}_{\text{y}}{\text{G}}^{\text{1}}{}_{\text{z}}{\text{T}}^{\text{2}}{}_{\text{x}}{\text{T}}^{\text{2}}{}_{\text{y}}{\text{G}}^{\text{2}}{}_{\text{z}}{\text{,,G}}^{\text{1}}{}_{\text{x}}{\text{T}}^{\text{1}}{}_{\text{y}}{\text{G}}^{\text{1}}{}_{\text{z}}{\text{G}}^{\text{2}}{}_{\text{x}}{\text{T}}^{\text{2}}{}_{\text{y}}{\text{T}}^{\text{2}}{}_{\text{z}}{\text{,G}}^{\text{1}}{}_{\text{x}}{\text{T}}^{\text{1}}{}_{\text{y}}{\text{G}}^{\text{1}}{}_{\text{z}}{\text{T}}^{\text{2}}{}_{\text{x}}{\text{G}}^{\text{2}}{}_{\text{y}}{\text{T}}^{\text{2}}{}_{\text{z}},\;\\ {\text{T}}^{\text{1}}{}_{\text{x}}{\text{G}}^{\text{1}}{}_{\text{y}}{\text{G}}^{\text{1}}{}_{\text{z}}{\text{T}}^{\text{2}}{}_{\text{x}}{\text{T}}^{\text{2}}{}_{\text{y}}{\text{G}}^{\text{2}}{}_{\text{z}}{\text{,T}}^{\text{1}}{}_{\text{x}}{\text{G}}^{\text{1}}{}_{\text{y}}{\text{G}}^{\text{1}}{}_{\text{z}}{\text{G}}^{\text{2}}{}_{\text{x}}{\text{T}}^{\text{2}}{}_{\text{y}}{\text{T}}^{\text{2}}{}_{\text{z}}{\text{,T}}^{\text{1}}{}_{\text{x}}{\text{G}}^{\text{1}}{}_{\text{y}}{\text{G}}^{\text{1}}{}_{\text{z}}{\text{T}}^{\text{2}}{}_{\text{x}}{\text{G}}^{\text{2}}{}_{\text{y}}{\text{T}}^{\text{2}}{}_{\text{z}},\;\\ {\text{T}}^{\text{1}}{}_{\text{x}}{\text{G}}^{\text{1}}{}_{\text{y}}{\text{T}}^{\text{1}}{}_{\text{z}}{\text{G}}^{\text{2}}{}_{\text{x}}{\text{G}}^{\text{2}}{}_{\text{y}}{\text{T}}^{\text{2}}{}_{\text{z}}{\text{,T}}^{\text{1}}{}_{\text{x}}{\text{G}}^{\text{1}}{}_{\text{y}}{\text{T}}^{\text{1}}{}_{\text{z}}{\text{T}}^{\text{2}}{}_{\text{x}}{\text{G}}^{\text{2}}{}_{\text{y}}{\text{G}}^{\text{2}}{}_{\text{z}}{\text{,T}}^{\text{1}}{}_{\text{x}}{\text{G}}^{\text{1}}{}_{\text{y}}{\text{T}}^{\text{1}}{}_{\text{z}}{\text{G}}^{\text{2}}{}_{\text{x}}{\text{T}}^{\text{2}}{}_{\text{y}}{\text{G}}^{\text{2}}{}_{\text{z}},\;\\ {\text{G}}^{\text{1}}{}_{\text{x}}{\text{T}}^{\text{1}}{}_{\text{y}}{\text{T}}^{\text{1}}{}_{\text{z}}{\text{G}}^{\text{2}}{}_{\text{x}}{\text{G}}^{\text{2}}{}_{\text{y}}{\text{T}}^{\text{2}}{}_{\text{z}}{\text{,G}}^{\text{1}}{}_{\text{x}}{\text{T}}^{\text{1}}{}_{\text{y}}{\text{T}}^{\text{1}}{}_{\text{z}}{\text{T}}^{\text{2}}{}_{\text{x}}{\text{G}}^{\text{2}}{}_{\text{y}}{\text{G}}^{\text{2}}{}_{\text{z}}{\text{,G}}^{\text{1}}{}_{\text{x}}{\text{T}}^{\text{1}}{}_{\text{y}}{\text{T}}^{\text{1}}{}_{\text{z}}{\text{G}}^{\text{2}}{}_{\text{x}}{\text{T}}^{\text{2}}{}_{\text{y}}{\text{G}}^{\text{2}}{}_{\text{z}}\} \end{array}$$

Subscripts above indicate clinics, and superscripts indicate patient order. For instance, the first action listed is G^1^_x_G^1^_y_T^1^_z_ T^2^_x_T^2^_y_G^2^_z_, which has the following meaning: assign control treatment to first patient at clinic X; assign control treatment to first patient at clinic Y; assign active treatment to first patient at clinic Z; assign active treatment to second patient at clinic X; assign active treatment to second patient at clinic Y; assign control treatment to second patient at clinic Z. Each of the eighteen sequences in C_stat_ results in acceptable overall trial imbalance at the end of the first day and acceptable overall trial imbalance at the end of the second day.

#### Investigators’ strategies and information partitions with three clinical sites

Each strategy for an investigator here consists of a probabilistic forecast before the first treatment has been unmasked and a probabilistic forecast after the first treatment is unmasked. We denote by I^1^_i_ investigator i’s information set when he chooses a treatment forecast for clinic i’s first patient, where i is any particular investigator, iϵ{x, y, z}. p_i_|h^1^_i_ is i's forecast for the first patient, with zero treatments previously unmasked. When making the first forecast, the investigator cannot distinguish between any members of C_stat_ having been selected.

Following the first treatments, each site investigator observes which treatment their first patient received. This unmasking allows each investigator to condition his forecast for the second patient’s treatment on his observation of the first patient’s treatment. When making the second forecast, p_i_|h^2^_i_, histories following the unmasking of an active first treatment at clinic i are distinguished from histories following the unmasking of a control first treatment at clinic i. We denote by I^T^_i_ investigator i’s information set following the former kind of histories, and we denote by I^G^_i_ investigator i's information set following the latter kind of histories. Each history in the information set I^T^_x_ begins with one of the following treatment sequences: T^1^_x_T^1^_y_G^1^_z_ G^2^_x_G^2^_y_T^2^_z_, T^1^_x_T^1^_y_G^1^_z_ T^2^_x_G^2^_y_G^2^_z_, T^1^_x_T^1^_y_G^1^_z_ G^2^_x_T^2^_y_G^2^_z_, T^1^_x_G^1^_y_G^1^_z_ T^2^_x_T^2^_y_G^2^_z_, T^1^_x_G^1^_y_G^1^_z_ G^2^_x_T^2^_y_T^2^_z_, T^1^_x_G^1^_y_G^1^_z_ T^2^_x_G^2^_y_T^2^_z_, T^1^_x_G^1^_y_T^1^_z_ G^2^_x_G^2^_y_T^2^_z_, T^1^_x_G^1^_y_T^1^_z_ T^2^_x_G^2^_y_G^2^_z_, or T^1^_x_G^1^_y_T^1^_z_ G^2^_x_T^2^_y_G^2^_z_. The site X investigator knows that the statistician has selected one of these sequences whenever he observes an active treatment for site X’s first patient, but he cannot distinguish which one of these was selected partly because he cannot observe the first treatments at sites Y and Z. It is commonly assumed that each clinical investigator knows the treatment history at his own site but it unaware of treatments assigned previously at other sites. See, for instance, [18], [19], [20], and [21]. As a result, investigator X is unaware of the overall treatment imbalance, although the site-specific imbalance may be imperfectly correlated with the overall imbalance. Each history in investigator X’s other information set, I^G^_x_, begins with one of the other treatment sequences, i.e. a sequence where site X’s first patient receives glycerin control.

Similarly, each history in the information set I^T^_y_ begins with one of the following treatment sequences: T^1^_x_T^1^_y_G^1^_z_ G^2^_x_G^2^_y_T^2^_z_, T^1^_x_T^1^_y_G^1^_z_ T^2^_x_G^2^_y_G^2^_z_, T^1^_x_T^1^_y_G^1^_z_ G^2^_x_T^2^_y_G^2^_z_, G^1^_x_T^1^_y_G^1^_z_ T^2^_x_T^2^_y_G^2^_z_, G^1^_x_T^1^_y_G^1^_z_ G^2^_x_T^2^_y_T^2^_z_, G^1^_x_T^1^_y_G^1^_z_ T^2^_x_G^2^_y_T^2^_z_, G^1^_x_T^1^_y_T^1^_z_ G^2^_x_G^2^_y_T^2^_z_, G^1^_x_T^1^_y_T^1^_z_ T^2^_x_G^2^_y_G^2^_z_, or G^1^_x_T^1^_y_T^1^_z_ G^2^_x_T^2^_y_G^2^_z_. The site Y investigator knows that the statistician has selected one of these sequences whenever he observes an active treatment for site Y’s first patient, but he cannot distinguish which one of these was selected partly because he cannot observe the first treatments at sites X and Z. Each history in investigator Y’s other information set, I^G^_y_, begins with a sequence where Y’s first patient receives control treatment.

Following the same logic for investigator Z, each history in the information set I^T^_z_ begins with one of the following treatment sequences: G^1^_x_G^1^_y_T^1^_z_ T^2^_x_T^2^_y_G^2^_z_, G^1^_x_G^1^_y_T^1^_z_ G^2^_x_T^2^_y_T^2^_z_, G^1^_x_G^1^_y_T^1^_z_ T^2^_x_G^2^_y_T^2^_z_, T^1^_x_G^1^_y_T^1^_z_ G^2^_x_G^2^_y_T^2^_z_, T^1^_x_G^1^_y_T^1^_z_ T^2^_x_G^2^_y_G^2^_z_, T^1^_x_G^1^_y_T^1^_z_ G^2^_x_T^2^_y_G^2^_z_, G^1^_x_T^1^_y_T^1^_z_ G^2^_x_G^2^_y_T^2^_z_, G^1^_x_T^1^_y_T^1^_z_ T^2^_x_G^2^_y_G^2^_z_, or G^1^_x_T^1^_y_T^1^_z_ G^2^_x_T^2^_y_G^2^_z_. The site Z investigator knows that the statistician has selected one of these sequences whenever he observes an active treatment for site Z’s first patient, but he cannot distinguish which one of these was selected partly because he cannot observe the first treatments at sites X and Y. Each history in investigator Z’s other information set, I^G^_z_, begins with a sequence where Z’s first patient receives control treatment.

#### Run-reversal equilibrium with three clinical sites

For a two-trio unmasked clinical trial with three clinical sites (site X, site Y, and site Z) and a maximum tolerable imbalance equal to one, a run-reversal equilibrium is shown in Table 1. A RRE for any three-site trial with more patients consists of repeated play of the strategies in Table 1. If 100 patients were enrolled at each site, then the RRE would consist of 50 repetitions of the strategies shown; 200 patients at each site would result in 100 repetitions of the strategies shown; etc.

**Table 1:** A Run-Reversal Equilibrium with Three Clinical Sites.

Player	Pure Strategy	Probability
statistician		
	G^1^_x_G^1^_y_T^1^_z_ T^2^_x_T^2^_y_G^2^_z_	1/24 = 0.04167
	T^1^_x_T^1^_y_G^1^_z_ G^2^_x_G^2^_y_T^2^_z_	1/24
	G^1^_x_T^1^_y_G^1^_z_ T^2^_x_G^2^_y_T^2^_z_	1/24
	T^1^_x_G^1^_y_G^1^_z_ G^2^_x_T^2^_y_T^2^_z_	1/24
	T^1^_x_G^1^_y_T^1^_z_ G^2^_x_T^2^_y_G^2^_z_	1/24
	G^1^_x_T^1^_y_T^1^_z_ T^2^_x_G^2^_y_G^2^_z_	1/24
	G^1^_x_G^1^_y_T^1^_z_ G^2^_x_T^2^_y_T^2^_z_	1/16 = 0.0625
	G^1^_x_G^1^_y_T^1^_z_ T^2^_x_G^2^_y_T^2^_z_	1/16
	T^1^_x_T^1^_y_G^1^_z_ T^2^_x_G^2^_y_G^2^_z_	1/16
	T^1^_x_T^1^_y_G^1^_z_ G^2^_x_T^2^_y_G^2^_z_	1/16
	G^1^_x_T^1^_y_G^1^_z_ G^2^_x_T^2^_y_T^2^_z_	1/16
	G^1^_x_T^1^_y_G^1^_z_ T^2^_x_T^2^_y_G^2^_z_	1/16
	T^1^_x_G^1^_y_G^1^_z_ T^2^_x_T^2^_y_G^2^_z_	1/16
	T^1^_x_G^1^_y_G^1^_z_ T^2^_x_G^2^_y_T^2^_z_	1/16
	T^1^_x_G^1^_y_T^1^_z_ T^2^_x_G^2^_y_G^2^_z_	1/16
	T^1^_x_G^1^_y_T^1^_z_ G^2^_x_G^2^_y_T^2^_z_	1/16
	G^1^_x_T^1^_y_T^1^_z_ G^2^_x_G^2^_y_T^2^_z_	1/16
	G^1^_x_T^1^_y_T^1^_z_ G^2^_x_T^2^_y_G^2^_z_	1/16
investigator X		
	p_x_(I^0^_x_) = .5, p_x_(I^T^_x_) = .5, p_x_(I^G^_x_) = .5	1
investigator Y		
	p_y_(I^0^_y_) = .5, p_y_(I^T^_y_) = .5, p_y_(I^G^_y_) = .5	1
investigator Z		
	p_z_(I^0^_z_) = .5, p_z_(I^T^_z_) = .5, p_z_(I^G^_z_) = .5	1

Proof: For each investigator i, each forecast in Table 1 equals the Bayesian posterior probability conditional on the statistician’s strategy μ* in Table 1. Deviating from the Bayesian prediction would reduce predictability and therefore each forecast profile in Table 1 is an investigator-best-response to μ*. Given the forecast profiles in Table 1, excess predictability equals zero and therefore the statistician’s payoff is maximized by the randomization procedure μ*. Thus there is no profitable deviation for any player.

#### Runs and reversals in treatment imbalance under the RRE

An attractive feature of RRE randomization here is that treatment imbalance follows a random walk at each clinic. The investigator at each clinic is therefore allowed minimal success at predicting his clinic’s treatments. This is true in spite of the fact that reversals in treatment imbalance are more likely than runs in treatment imbalance for the overall trial.

Whenever one treatment has been assigned more frequently than another, let the imbalance IMB_i,t_ equal the number of times that T has previously been assigned minus the number of times that G has previously been assigned at clinic i. The t-th treatment at clinic i will either increase the clinic’s imbalance by 1 or decrease the clinic’s imbalance by 1, as denoted by the following imbalance indicator variable:

$$\begin{array}{l} RUN_{i,t}\left(c_{stat}\right)=1\;if\;IMB_{i,t}>IMB_{i,t-1}>0\;OR\;if\;IMB_{i,t}<\;IMB_{i,t-1}<0\\ RUN_{i,t}\left(c_{stat}\right)=0\;if\;IMB_{i,t}<IMB_{i,t-1}>0\;OR\;if\;IMB_{i,t}>\;IMB_{i,t-1}<0 \end{array}$$ (6)

RUN_i,t_ = 1 if the t-th treatment in the sequence c_stat_ played by the statistician exacerbates the clinic’s imbalance, i.e. if there is a “run” in imbalance. RUN_i,t_ = 0 if the t-th treatment in the sequence c_stat_ played by the statistician mitigates the clinic’s imbalance, i.e. if there is a “reversal” in imbalance. In choosing how to forecast treatments, the investigator compares the likelihood of runs to the likelihood of reversals. Across all possible treatment sequences, the likelihood of treatment runs equals the summation of μ_stat_(c_stat_)·RUN_i,t_(c_stat_), where μ_stat_(c_stat_) is the probability that the statsitician plays the sequence c_stat_. Similarly, the likelihood of treatment reversals equals the summation of μ_stat_(c_stat_)·[1−RUN_i,t_(c_stat_)].

#### Definition of the random walk property of RRE for clinical trial randomization

When each clinic’s treatment imbalance is unconstrained, any RRE satisfies:

$$SUM\;\mu_{stat}\left(c_{stat}\right)\cdot RUN_{i,t}\left(c_{stat}\right)=\;\frac{1}{2}=\;SUM\;\mu_{stat}\left(c_{stat}\right)\cdot \left[1-RUN_{i,t}\left(c_{stat}\right)\right]$$ (7)

That is, when each clinic’s treatment imbalance is unconstrained, any RRE makes runs in treatment imbalance at any particular clinic as equally likely as reversals in treatment imbalance.

**Fig 1:**
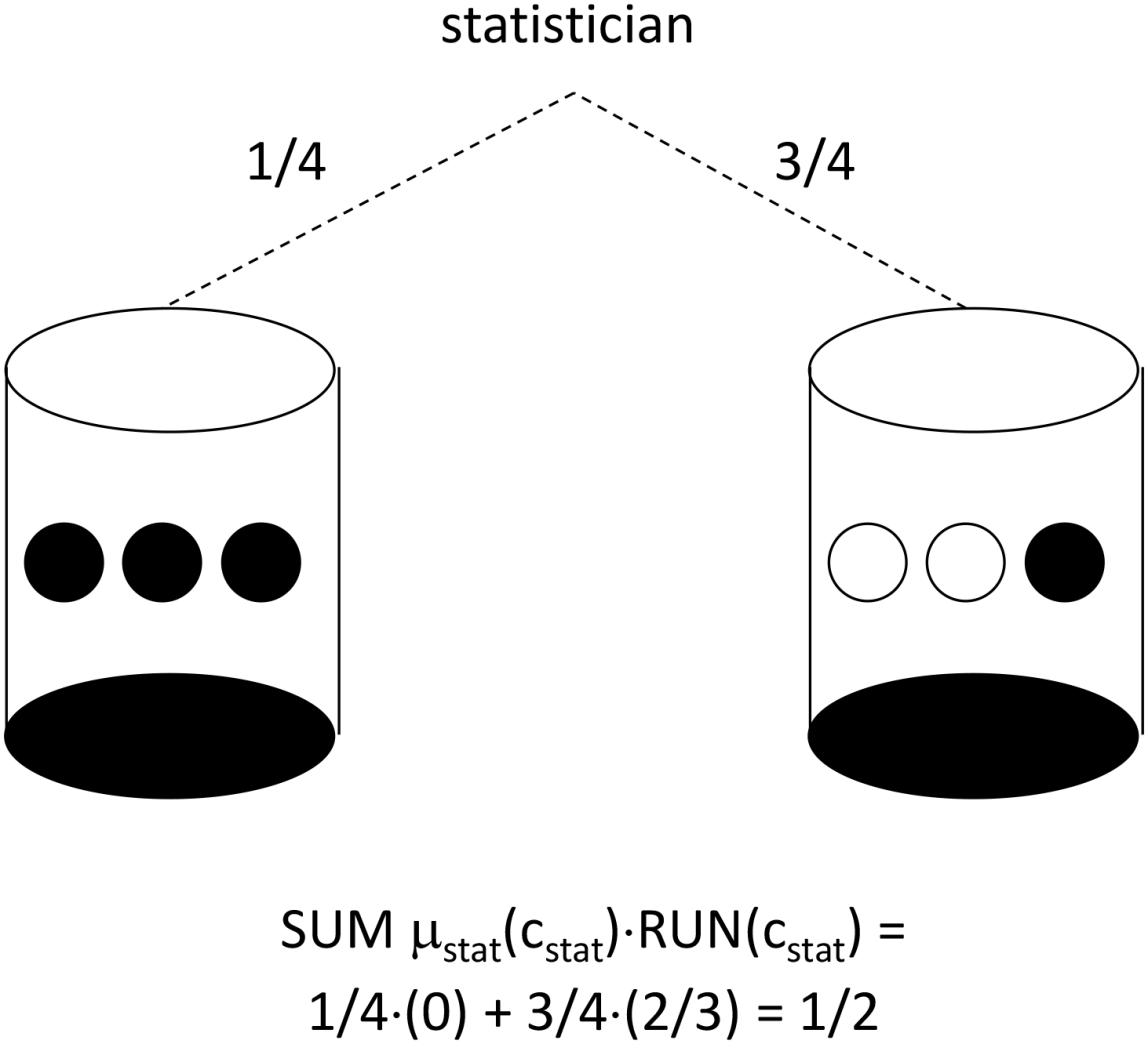
Urn Design for Run-Reversal Equilibrium with 3 Clinics. An urn is selected according to the probabilities shown, and then balls are drawn without replacement until the urn is empty. A run in treatment imbalance is assigned for a clinic when a white ball is drawn. A reversal in treatment imbalance is assigned for a clinic when a black ball is drawn.

The random-walk property can be depicted with repeated draws from urns of black and white balls. For the RRE derived in section 2.3, Fig 1 shows two urns, each of which is filled with three balls. Imagine that the statistician first chooses one of the urns, and subsequently, the statistician selects a clinic-x ball from the chosen urn, then selects a clinic-y ball from the same chosen urn (without replacing the first ball), and then draws a final clinic-z ball from the same chosen urn (without replacing the first two balls). Ball color indicates whether or not a clinic’s treatment imbalance gets reversed. Each clinic assigned a white ball experiences a run in treatment imbalance, while each clinic assigned a black ball experiences a reversal in treatment imbalance.

Because there are three clinics, there are exactly two possibilities:

- all three clinics will experience reversals in treatment imbalance, with zero clinics experiencing runs in treatment imbalance (the left-hand urn in Fig 1 is selected)

OR

- one clinic will experience a reversal in treatment imbalance, with two clinics experiencing runs in treatment imbalance (the right-hand urn in Fig 1 is selected)

One of the two urns must be selected, in order to restore perfect balance for the overall trial. Conditional on the left-hand urn being selected, each clinic faces a probability of black ball equal to 100%. Conditional on the right-hand urn being selected, each clinic faces a probability of black ball equal to 33%. According to the NE distribution over treatment sequences, the left-hand urn is selected with probability ¼ (each of the first six sequences in Table 1 corresponds to the left-hand urn, and each of these six sequences receives probability 1/24), while the right-hand urn is selected with probability ¾ (each of the last twelve sequences in Table 1 corresponds to the right-hand urn, and each of these twelve sequences receives probability 1/16).

The RRE places greater probability on the right-hand urn because of a special type of uncertainty: conditional on selection of the right-hand urn, each investigator does not know which clinic will experience imbalance reversal. The statistician plays a treatment sequence where all three clinics experience reversal (the left-hand urn) with 25% probability, and conditional on one such sequence being selected, each investigator knows it is 100% likely that their clinic will experience reversal. The statistician plays a sequence where only one clinic experiences reversal (the right-hand urn) with 75% probability, and conditional on one such sequence being selected, each investigator knows it is 33% likely that their clinic will experience reversal.

The result is each investigator attaches probability equal to ¼(1) + ¾(1/3) = ½ on the possibility of imbalance reversal, while attaching probability equal to ¼(0) + ¾(2/3) = ½ on the possibility of a run in imbalance. The random-walk property may be restated by saying that any NE gives each clinic 50%-50% chances of black ball or white ball. The treatment imbalance thus follows a random walk from the perspective of each clinic’s investigator, while the overall trial’s treatment imbalance is reversed with 100% probability.

### Run-Reversal Equilibria with Four, Five, and Six Clinical Sites

#### A four-site RRE

**Fig 2:**
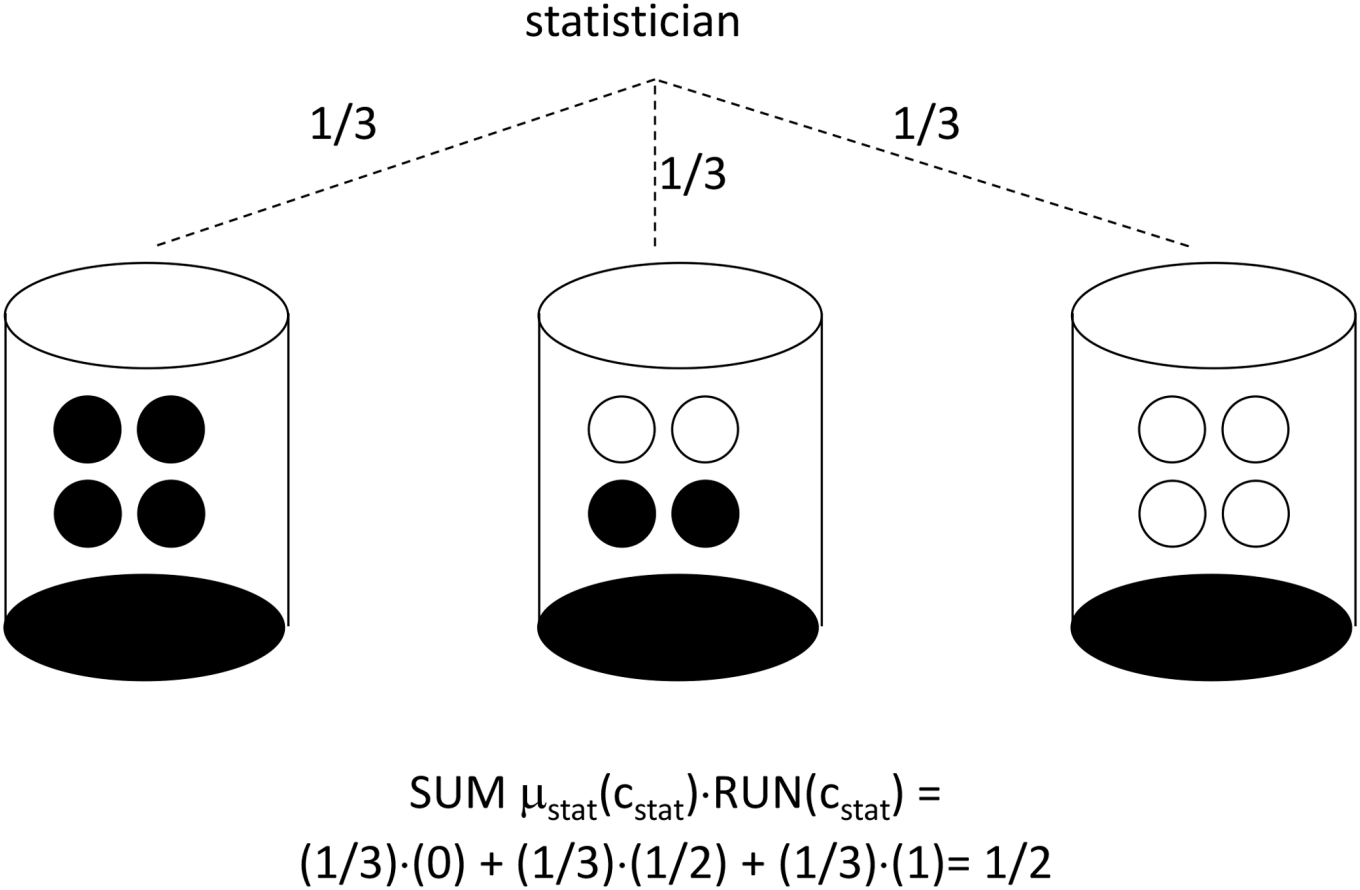
Urn Design for Run-Reversal Equilibrium with 4 Clinics. An urn is selected according to the probabilities shown, and then balls are drawn without replacement until the urn is empty. A run in treatment imbalance is assigned for a clinic when a white ball is drawn. A reversal in treatment imbalance is assigned for a clinic when a black ball is drawn.

Run-reversal equilibria can be computed for clinical trials with any number of sites. The number of treatment sequences and forecast profiles will grow much larger as the number of sites increases, but the nature of any RRE can easily be seen using the same kind of urn perspective introduced in section 4. For a four-site clinical trial, for instance, Fig 2 shows urns containing four balls, each of which will be drawn and used to determine whether or not treatment imbalance is reversed at each of the four clinics. Once again, a black ball indicates RUN_i,t_ = 0 (treatment imbalance is reversed) and a white ball indicates RUN_i,t_ = 1 (treatment imbalance is exacerbated). In order to restore perfect treatment balance for the overall trial, there are exactly three possibilities:

- all four clinics will experience reversals in treatment imbalance, with zero clinics experiencing runs in treatment imbalance (the left-hand urn in Fig 2 is selected)

OR

- two out of four clinics will experience reversals in treatment imbalance, with two clinics experiencing runs in treatment imbalance (the middle urn in Fig 2 is selected)

OR

- zero clinics will experience reversal in treatment imbalance, i.e. all four clinics will experience runs in treatment imbalance (the right-hand urn in Fig 2 is selected)

For the RRE corresponding to Fig 2, the statistician chooses the left urn with probability 1/3, the middle urn with probability 1/3, and the right urn with probability 1/3. Conditional on selection of the left urn, middle urn, and right urn, each investigator knows it is, respectively, 100%, 50%, and 0% likely that their clinic will experience reversal. The result is each investigator attaching probability equal to 1/3(100%) + 1/3(50%) + 1/3(0%) = ½ on the possibility of imbalance reversal, while attaching probability equal to 1/3(0%) + 1/3(50%) + 1/3(100%) = ½ on the possibility of a run in imbalance. Here again, the treatment imbalance follows a random walk from the perspective of each clinic’s investigator, while the overall trial’s treatment imbalance is reversed with 100% probability.

#### A five-site RRE

**Fig 3:**
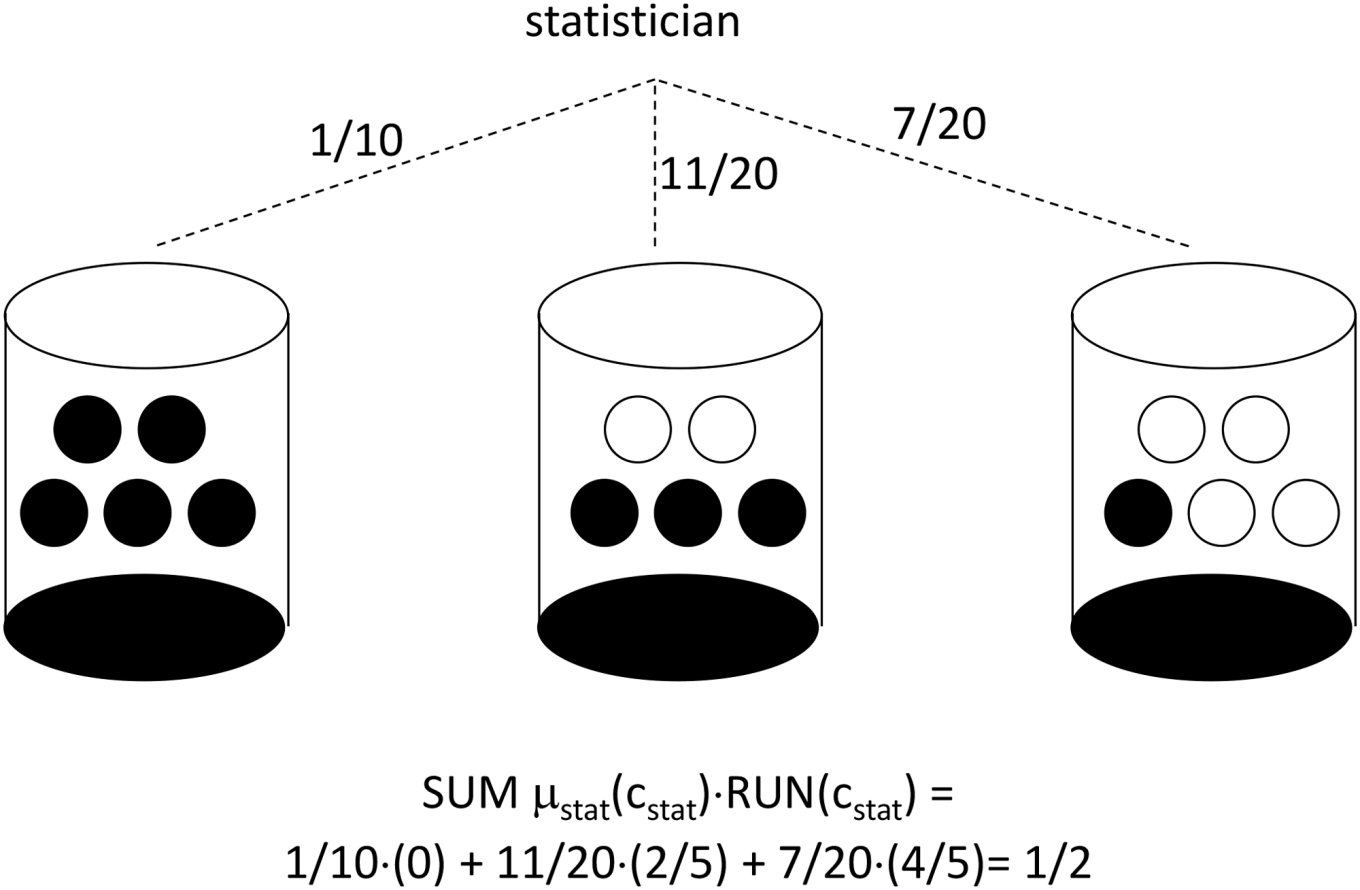
Urn Design for Run-Reversal Equilibrium with 5 Clinics. An urn is selected according to the probabilities shown, and then balls are drawn without replacement until the urn is empty. A run in treatment imbalance is assigned for a clinic when a white ball is drawn. A reversal in treatment imbalance is assigned for a clinic when a black ball is drawn.

Clinical trials with odd numbers of sites have RRE with less symmetry. For a five-site clinical trial, each urn in Fig 3 contains five balls, each of which will be drawn and used to determine whether or not treatment imbalance is reversed at each of five clinics. Once again, a black ball indicates RUN_i,t_ = 0 (treatment imbalance is reversed) and a white ball indicates RUN_i,t_ = 1 (treatment imbalance is exacerbated). In order to restore perfect treatment balance for the overall trial, there are exactly three possibilities:

- all five clinics will experience reversals in treatment imbalance, with zero clinics experiencing runs in treatment imbalance (the left-hand urn in Fig 3 is selected)

OR

- three out of five clinics will experience reversals in treatment imbalance, with two clinics experiencing runs in treatment imbalance (the middle urn in Fig 3 is selected)

OR

- a single clinic will experience reversal in treatment imbalance, with the other four clinics experiencing runs in treatment imbalance (the right-hand urn in Fig 3 is selected)

For the RRE corresponding to Fig 3, the statistician chooses the left urn with probability 1/10, the middle urn with probability 11/20, and the right urn with probability 7/20. Conditional on selection of the left urn, middle urn, and right urn, each investigator knows it is, respectively, 100%, 60%, and 20% likely that their clinic will experience reversal. The result is each investigator attaching probability equal to1/10(100%) + 11/20(60%) + 7/20(20%) = ½ on the possibility of imbalance reversal, while attaching probability equal to 1/20(0%) + 11/20(40%) + 7/20(80%) = ½ on the possibility of a run in imbalance. Here again, the treatment imbalance follows a random walk from the perspective of each clinic’s investigator, while the overall trial’s treatment imbalance is reversed with 100% probability.

#### A six-site RRE

**Fig 4:**
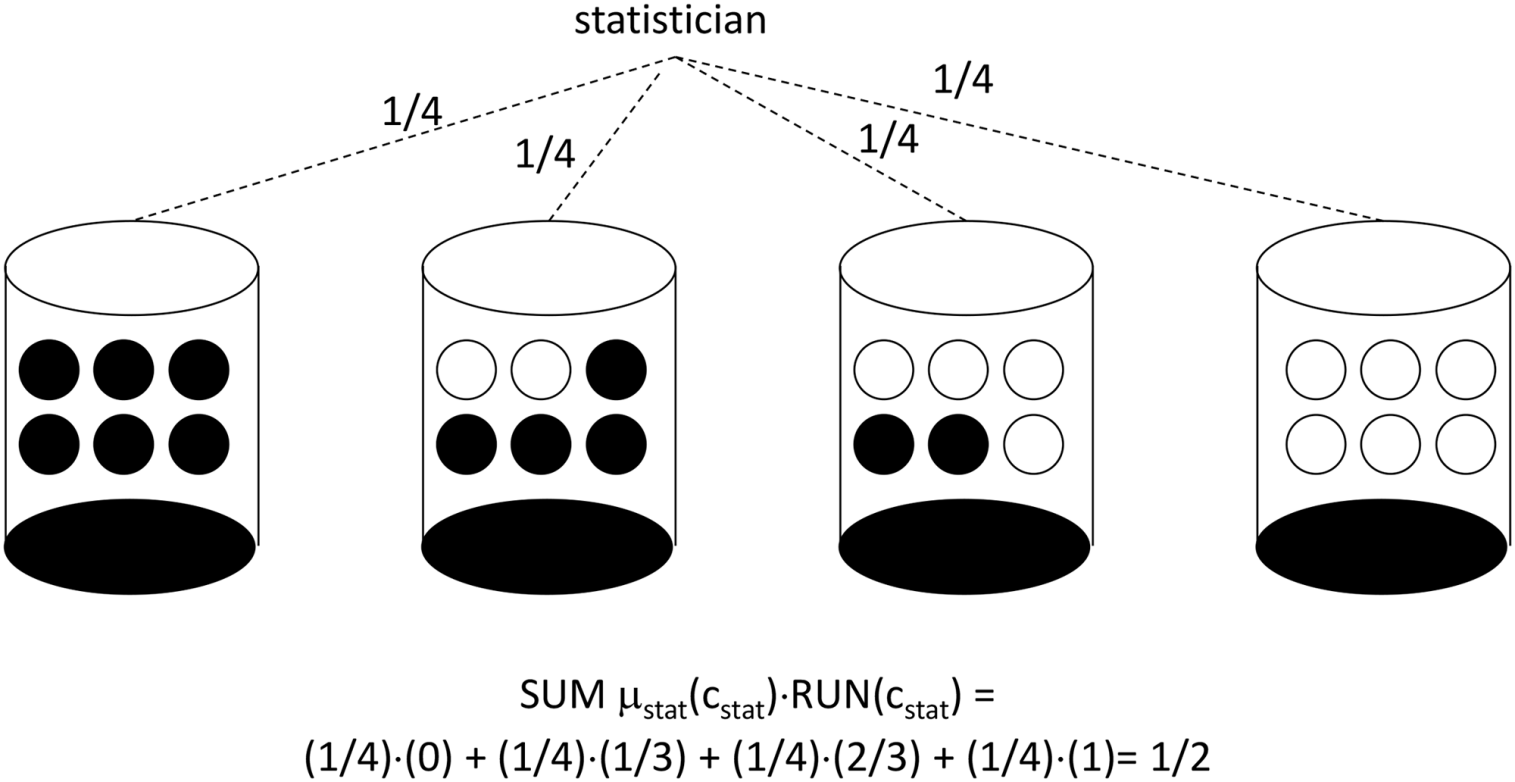
Urn Design for Run-Reversal Equilibrium with 6 Clinics. An urn is selected according to the probabilities shown, and then balls are drawn without replacement until the urn is empty. A run in treatment imbalance is assigned for a clinic when a white ball is drawn. A reversal in treatment imbalance is assigned for a clinic when a black ball is drawn.

For a six-site clinical trial, each urn in Fig 4 contains six balls, each of which will be drawn and used to determine whether or not treatment imbalance is reversed at each of six clinics, again with a black ball indicating RUN_i,t_ = 0 (treatment imbalance is reversed) and a white ball indicating RUN_i,t_ = 1 (treatment imbalance is exacerbated). In order to restore perfect treatment balance for the overall trial, there are exactly four possibilities:

- all six clinics will experience reversals in treatment imbalance, with zero clinics experiencing runs in treatment imbalance (the far left urn in Fig 4 is selected)

OR

- four out of six clinics will experience reversals in treatment imbalance, with two clinics experiencing runs in treatment imbalance (the second urn in Fig 4 is selected)

OR

- two out of six clinics will experience reversals in treatment imbalance, with the other four clinics experiencing runs in treatment imbalance (the third urn in Fig 4 is selected)

OR

- zero clinics will experience reversal in treatment imbalance, i.e. all six clinics will experience runs in treatment imbalance (the far right urn in Fig 4 is selected)

For the RRE corresponding to Fig 4, the correlation device recommends each of the four urns with probability 1/4. Conditional on selection of the first, second, third or fourth urn, each investigator knows it is, respectively, 100%, 66%, 33%, and 0% likely that *their clinic* will experience reversal. The result is each investigator attaching probability equal to 1/4(100%) 1/4(66%) + 1/4(33%) + 1/4(0%) = ½ on the possibility of imbalance reversal, while attaching probability equal to 1/4(0%) 1/4(33%) + 1/4(66%) + 1/4(100%) = ½ on the possibility of a run in imbalance. Here again, the treatment imbalance follows a random walk from the perspective of each clinic’s investigator, while the overall trial’s treatment imbalance is reversed with 100% probability.

## Conclusions

Randomization procedures based on run-reversal equilibrium offer a new and improved way to minimize treatment predictability in clinical trials. The RRE method improves upon variable block randomization, for example, because RRE thwarts simplistic guessing strategies that are imprecise yet problematic for variable block methods. Supposed virtues of variable-block randomization are that investigators are highly unlikely to know the true probability of the next allocation, and also that variable-block randomization sometimes makes investigators unaware when a deterministic allocation is next in the sequence. In spite of these qualities, investigators can nonetheless follow very simple guessing strategies to create bias under variable-block randomization. One example is the convergent strategy, which is when the investigator always guesses that the next allocation will be whichever treatment has previously been allocated less frequently. Another example is the reverse-the-last guessing strategy, which is when the investigator guesses that the next allocation will be the opposite of whichever treatment was allocated most recently. While requiring no probabilistic calculation by the investigator, each of these guessing strategies is highly likely to create excess predictability and potential bias against variable-block randomization. Against the RRE method, these or other simplistic guessing strategies result in zero excess predictability and therefore minimal potential bias.

The practical use of RRE randomization procedures does, however, have some limitations. First of all, the game model presented here is not appropriate if investigators learn treatment histories at sites other than their own. Our assumption that unmasking is own-site-specific is usually realistic, but if this assumption is violated, then the advantages of our randomization procedures are nullified. Also, not all trials have multiple sites. The game model presented here and the corresponding RRE are only applicable to multi-site experiments.

A more serious limitation is that some trials may wish to impose exactly the same imbalance constraints on each individual site as are imposed on the overall trial. Certainly, there will be some circumstances where it is desirable to impose perfect treatment balance at each site, in which case the randomization procedures presented here cannot be used. For instance, a trial may include different sites that have varying levels of practitioner experience, which could affect treatment outcomes, and therefore complete site-specific treatment balance might be needed. However, conditions requiring site-specific treatment balance are not always present and, in fact, they seem to only occur in a minority of trials. In a sample of 337 multi-site trials requiring treatment balance for the overall trial, for example, McEntegart reports that only 40 of these imposed treatment balance at each site [22]. A separate study, examining the Lancet, the British Medical Journal, and the New England Journal of Medicine, finds that less than 40% of intentionally unmasked clinical trials stratify randomization by site [21].

An additional concern is that stratifying by additional variables could complicate the RRE procedure. The main complication is added likelihood of overall (but not within-group) treatment imbalance. If one group has higher numbers of patients than another, then there is a greater likelihood that the RRE method will produce an imbalance in the treatments for the overall trial. Increasing the number of variables upon which the trial is stratified increases the chances that this occurs. As compared to minimization procedures, the RRE method and a minimization procedure are both capable of satisfying only two of three desirable goals:

1. achieving treatment balance within any number of subgroups (such as gender, race, etc),
2. achieving treatment balance for the overall trial regardless of variation in the numbers of patients in the different subgroups, and 3) achieving zero treatment predictability. Minimization methods achieve objectives 1 and 2, but they fail to achieve objective 3. The RRE method achieves objective 1 and 3, but it fails to achieve objective 2. The unavoidable reality of any restricted randomization procedure is a tradeoff between reducing predictability and reducing imbalance. Greater reduction in predictability will always entail some kind of greater chances of imbalance, and vice versa.

Depending upon the scale and scope of the trial, it may be desirable that, for a trial requiring perfect treatment balance for a large number of stratification variables, the RRE method might well be combined with a minimization method. Some centers could be randomized by RRE, and others randomized by a minimization method, and the overall trial results would be less vulnerable to the various kinds of bias that predictability and imbalance can produce.

In conclusion, we strongly agree with the common perception among clinical trial practitioners that predictability can be reduced by the inclusion of multiple sites [18], [19], [22]. However, we believe that existing randomization procedures fail to achieve this fully. The predictability of the randomization procedures most commonly used in RCTs has been quantified by numerous sources [12], [2], [10], [4], [15]. A run-reversal equilibrium distribution results in less predictability than any randomization methods currently in use for balance-constrained multi-site clinical trials, and is more realistic to implement than other, more informationally demanding game solutions. RRE procedures such as those described here are appropriate as long as treatment balance is needed for the overall trial but site-specific treatment balance is needed less or not at all. For clinical trials with these characteristics, a run-reversal equilibrium describes how a statistician can exploit the imperfect information that is created by a multiplicity of clinical sites for the purposes of reducing treatment predictability.
